# Accuracy of the Euro CTO(CASTLE) score obtained on coronary computed tomography angiography for Predicting 30-minute wire crossing in chronic total occlusions

**DOI:** 10.1186/s12872-022-02627-4

**Published:** 2022-04-19

**Authors:** Yan-tan Yu, Zhi-yi Sha, Shu-min Chang, Du-tian Zhai, Xiao-jiao Zhang, Ai-jie Hou, Wen-jie Feng, Dao-wei Li, Yong Wang, Bo Luan

**Affiliations:** 1 School of Graduate, Dalian Medical University, Lushunkou District, No. 9, West Section of Lushun South Road, Dalian, 116041 China; 2 Department of Cardiology, The People’s Hospital of China Medical University, People’s Hospital of Liaoning Province, No. 33, Wenyi Road, Shenhe District, Shenyang, 110000 China; 3 Department of Radiology, The People’s Hospital of China Medical University, People’s Hospital of Liaoning Province, No. 33, Wenyi Road, Shenhe District, Shenyang, 110000 China

**Keywords:** Coronary chronic total occlusion, Coronary angiography, Percutaneous coronary intervention, Computed tomography angiography

## Abstract

**Background:**

To investigate the feasibility and accuracy of the Euro CTO (CASTLE)_CTA_ score obtained on coronary computed tomography angiography (CCTA) for predicting the success of percutaneous coronary intervention (PCI) and the 30-min wire crossing in chronic total occlusions (CTO).

**Method:**

One hundred and fifty patients (154 CTO cases; median age, 61 (interquartile range [IQR], 54–68) years; 75.3% male) received CCTA at the People's Hospital of Liaoning Provincce within 1 month before the procedure. The Euro CTO (CASTLE) score obtained on CCTA(CASTLE_CTA_) was calculated and compared with the Euro CTO (CASTLE) score obtained based on coronary angiography (CASTLE_CAG_) for the predictive value of 30-min wire crossing and CTO procedural success.

**Results:**

In our study, the CTO-PCI success rate was 89.0%, with guidewires of 65 cases (42.2%) crossing within 30 min. There were no significant differences in the median CASTLE_CTA_ and CASTLE_CAG_ scores in the procedure success group (3 [IQR, 2–4] vs 3 (IQR, 2–3]; p = 0.126). However, the median CASTLE_CTA_ score was significantly higher than the median CASTLE_CAG_ score in the procedure failure group (4 [IQR, 3–5.5] vs 4 [IQR, 2.5–5.5]; p = 0.021). There was no significant difference between the median CASTLE_CTA_ score and the median CASTLE_CAG_ score in the 30-min wire crossing failure group (3 [IQR, 3–4] vs 3 [IQR, 2–4]; p = 0.254). However, the median CASTLE_CTA_ score was significantly higher than the median CASTLE_CAG_ score in the 30-min wire crossing group (3 [IQR, 2–3] vs 2 [IQR, 2–3]; p < 0.001). The CASTLE_CTA_ score described higher levels of calcification than the CASTLE_CAG_ score (48.1% vs 33.8%; p = 0.015). There was no significant difference between the CASTLE_CTA_ score (area under the curve [AUC], 0.643; 95% confidence interval [CI], 0.561–0.718) and the CASTLE_CAG_ score (AUC, 0.685; 95% CI, 0.606–0.758) for predicting procedural success (p = 0.488). The CASTLE_CTA_ score (AUC, 0.744; 95% CI, 0.667–0.811) was significantly better than the CASTLE_CAG_ score (AUC, 0.681; 95% CI, 0.601–0.754; p = 0.046) for predicting 30-min wire crossing with the best cut-off value being CASTLE_CTA_ ≤ 3. The sensitivity, specificity, positive predictive value, and negative predictive value were 90.8%, 55.2%, 54.6%, and 87.0%, respectively.

**Conclusion:**

The CASTLE_CTA_ scores obtained from noninvasive CCTA perform better for the prediction of the 30-min wire crossing than the CASTLE_CAG_ score.

## Background

Coronary chronic total occlusion (CTO) is defined as a coronary occlusion lesion for ≥ 3 months with no distal antegrade contrast passage (thrombosis in myocardial infarction grade 0). Studies have confirmed that 15–25% of patients undergoing coronary angiography (CAG) had CTO in the coronary arteries [1]. Among patients undergoing percutaneous coronary intervention (PCI), 10–20% had CTO, yet less than 8% underwent CTO revascularization [2]. The success rate gradually improved with the accumulation of operator experiencer, guidewire advancement technology, and related device improvement [3]. However, PCI for CTO lesions in the coronary arteries remains technically challenging and one of the most difficult areas of interventional cardiology.

An increasing number of scholars have attempted to assess CTO-PCI complexity using CTO scores obtained on coronary angiography to develop an appropriate CTO-PCI strategy. The preoperative assessment of CTO lesion characteristics helps grade CTO-PCI difficulty, thus optimizing clinical patient management and improving procedural success [4, 5]. With the widespread application of coronary CTA, the preoperative identification of CTO lesion characteristics by noninvasive coronary computed tomography (CT) angiography (CCTA) may inform the choice of interventional treatment strategy.

Despite the lack of systematic comparisons of the CCTA features between acute and chronic coronary total occlusion cases, acute occlusion usually manifests as pronounced positive remodeling and the absence of extensive calcifications, whereby the distal vessel segment may lack clear contrast opacification via the collateral circulation [6]**.** Compared with invasive angiography, coronary CTA may enable full visualization of the occlusion site and distal vessels [7–9]. CCTA had a distinct advantage in the evaluation of long tortuous CTO lesions and the distal segments of CTO lesions [10]. However, it is unclear whether the Euro CTO (CASTLE) score obtained on CCTA(CASTLE_CTA_) can predict technical failure and 30-min wire crossing success rate. Therefore, this study aimed to investigate the predictive value of the CASTLE_CTA_ score obtained by CCTA versus the CASTLE_CAG_ score obtained on coronary angiography to verify their feasibility and accuracy for predicting CTO-PCI procedure and 30-min guidewire crossing success.

## Method

### Study population

This single-center retrospective observational study included 150 patients (154 CTO lesions; median age, 61 [54–68] years; 75.3% men) who underwent CCTA within 1 month prior to CAG at the People's Hospital of Liaoning Provincce between January 2016 and January 2021. The exclusion criteria were no CCTA examination within 1 month before CTO-PCI at our center; CCTA images with grade 3 motion artifacts; documented allergy to iodinated contrast; renal failure (glomerular filtration rate < 45 ml/min/1.73 m^2^); and previous stent placement at the occlusion site. Baseline information and previous medical history were obtained from each patient’s medical record. All subjects signed an informed consent form.

### Catheter angiography and CASTLE score analysis

The EuroCTO (CASTLE) score study [11] analyzed data from the EuroCTO registry. This prospective database was set up in 2008 and includes > 20,000 cases submitted by CTO expert operators (> 50 cases/year). Derivation (n = 14,882) and validation (n = 5745) datasets were created to develop a risk score for predicting technical failure. A total of six predictors were associated with procedural failure, The 6 factors above together formed a 6-scale scoring tool, including Coronary artery bypass grafting history,age (≥ 70 years), stump anatomy (blunt or invisible), tortuosity degree (severe or unseen), length of occlusion (≥ 20 mm), and extent of calcification (severe), each factor was assigned 1 point. The morphology of the vessel stump was classified as tapered, blunt, or unseen according to its appearance on fluoroscopy. Tortuosity degree were defined as straight (the pre-occlusive segment contained a bend of < 70°); moderate (a segment containing 2 bends > 70° or 1 bend > 90°); severe (CTO vessel contained 2 or more pre-occlusive bends > 90° or at least 1 bend > 120°).The length of coronary occlusions was estimated from angiographic projections visually with single- or dual-contrast injections. The degree of calcification was visually estimated on fluoroscopy: moderate (half of the total CTO segment had visible residues); severe (extension of calcification to > 50% of the segment).

According to wiring direction (antegrade and retrograde) and whether or not the subintimal space was used (wiring versus dissection and re-entry), CTO crossing strategies were classified as antegrade wire escalation; antegrade dissection-re-entry; retrograde wire escalation or retrograde dissection-re-entry. The "hybrid" strategy combined two or more two technic above. In our study, all the patients firstly received antegrade PCI, if that fails, we try other technic. Therefore we classify the final strategy as antegrade and hybrid. All the procedure were performed by Luan Bo (corresponding author). The CASTLE_CAG_ score was independently calculated by two coronary intervention specialists with extensive experience using CAG data without knowledge of the CCTA data. In cases of interobserver disagreement, consensus was reached after discussion.

### CTA protocol and CASTLE score analysis

A 256-slice CT scanner (Revolution CT, GE Healthcare) was used for imaging. All the CCTA data showed diagnostic quality, and they were included for further analysis.The image data were reconstructed using a 256-slice CT scanner(0.625 mm section thickness, 0.4 mm increment). Multi-planar reconstructions (MPRs), maximum intensity projections, and curved precision-recall (CPR) were obtained. CCTA post-processing was performed on a dedicated workstation (Vitrea, Canon). For all CTOs, the vessel lumen and vessel diameters were measured on orthogonal images, and the lengths were measured on CPR or MPRs. Imaging and evaluation criteria were based on the CASTLE scoring system. The images were read by two cardiovascular imaging diagnosticians with extensive experience (associate physician or higher title). The CTO lesions were diagnosed and analyzed independently using a double-blind reading approach with blinding of each patient's clinical information and previous examination findings. The CCTA-derived CASTLE_CTA_ score was calculated. In cases of interobserver disagreement, a consensus was reached after discussion.

### Statistical analysis

Two endpoints were established in this study, namely procedural success and successful wire crossing within 30 min, with the primary focus being the latter [8, 12–15] since it is a more historically objective index of procedural difficulty. Procedural success was defined as a residual stenosis of < 10% at the end of the procedure with TIMI flow grade 3 antegrade flow. Wire manipulation time is the time from initial insertion of the wire into the coronary lumen to the time it was successfully crossed through the lesion or was pulled out of the lumen because of unsuccessful wire crossing. All statistical analyses were performed using SPSS 20.0. Due to the non-normal distribution of the data, dichotomous variables are expressed as numbers using percentages (%), while continuous variables are expressed as median and IQR. To compare the CASTLE scores obtained from CAG and CCTA, CASTLE_CAG_ scores and CASTLE_CTA_ scores were used as continuous variables. The continuous variables were compared using the Mann–Whitney U test and the Wilcoxon test, while categorical variables were compared using McNemar’s test. The predictive values of the CASTLE_CTA_ score and the CASTLE_CAG_ score were assessed using the subject operating characteristics (ROC) analysis by calculating the corresponding AUC. The method of DeLong et al.was used to compare the ROC curves. The optimal cut-off value to predict successful wire crossing within 30 min was determined using Youden's index. Statistical significance was set at values of p ≤ 0.05.

## Results

### Baseline characteristics and procedural data

A total of 150 patients (154 CTO lesions; median age, 61 [54–68] years; 75.3% male) were included; of them, 24 were ≥ 70 years old (16%), 19 (12.7%) had a history of coronary artery bypass grafting (CABG), 62 (41%) had a history of myocardial infarction, and 100 (67%) had a history of PCI. 76(49%) used intravascular ultrasound (IVUS). Detailed patient baseline information is shown in Table 1.

**Table 1:** Patients’ baseline data and procedural data

	Total
Age (years)	61 (54–68)
Sex (male/female)	113/37
Body mass index	26 (24–27)
Diabetes	44 (29)
Hypertension	90 (60)
Prior MI	62 (41)
Prior PCI	100 (67)
Prior CABG	19 (12.7)
Current smoker	76 (51)
Echocardiographic, LVEF%	0.45 (0.41–0.55)
LDL-C, mmol/l	2.17 (1.74–2.56)
IVUS	76 (49)
Contrast volume, ml	230 (167.5–330)
Procedural time, min	89 (63–125)

Data are presented as median (interquartile range) or number (%).MI, myocardial infarction; PCI, percutaneous coronary intervention; CABG, coronary artery bypass grafting; LVEF, left ventricular ejection fraction; LDL-C, low-density lipoprotein cholesterol; IVUS, intravascular ultrasound;

### Angiographic and procedural characteristics

Procedural success was achieved in 137 cases (89.0%), while wire crossing was achieved within 30 min in 65 cases (42.2%). Antegrade approach includes antegrade wire escalation or antegrade dissection re-entry, 154 individuals were included in this study, all the patients firstly received antegrade PCI and 55 patients successfully completed antegrade wire crossing within 30 min and 32 patients over 30 min completed antegrade wire crossing. Only 7 patients completed reverse-Cart and completed antegrade wire crossing within 30 min. The majority of patients (55) received reverse-Cart after antegrade PCI failure and completed antegrade wire crossing over 30 min. The median J-CTO score for all lesions was 3 (IQR, 3–4), with higher J-CTO scores in the procedural failure group than in the procedural success group (4.5 [IQR, 4–5] vs 3 [IQR, 3–4]; p < 0.001) and higher J-CTO scores in patients in the 30-min wire crossing failure group than in those in the 30-min wire crossing group (4 [IQR, 3–4] vs 3 [IQR, 3–3]; p < 0.001). Further characteristics of the CTO lesions and procedural results are shown in Table 2.

**Table 2:** CTO lesion characteristics and procedural data

Lesion characteristics	Total	Procedural results			Guidewire crossing time		
		Success n = 137	Fail n = 17	*p*	< 30 min n = 65	> 30 min n = 89	*p*
J-CTO score	3 (3–4)	3 (3–4)	4.5 (4–5)	< 0.001	3 (3–3)	4 (3–4)	< 0.001
Target vessel				< 0.001			0.049
RCA	73 (47)	68 (44)	5 (3)		37 (24)	36 (23)	
LAD	65 (42)	60 (39)	5 (3)		20 (13)	45 (29)	
LCX	16(10)	9 (6)	7 (4)		8 (5)	8 (5)	
Previous failed lesion	42 (27)	36 (23)	6 (4)	0.405	11 (7)	31 (20)	0.017
Antegrade*	87 (56)	83 (53)	4 (3)		55 (36)	32 (21)	
Hybrid	67 (44)	54 (35)	13 (8)		10 (7)	57 (37)	
Reverse-CART	62 (40)	49 (31)	13 (8)		7 (4)	55 (36)	

Data are presented as median (interquartile range) or number (%) of a total 154 lesions (%). CTO, chronic total occlusion; J-CTO, Multicenter CTO Registry of Japan; LAD, left anterior descending branch; LCX, left circumflex branch; RCA, right coronary artery

*Antegrade includes antegrade wire escalation or antegrade dissection re-entry. CART, controlled antegrade and retrograde tracking;

### Comparison of CASTLE scores derived from CCTA and CAG in procedural results

**Fig. 1:**
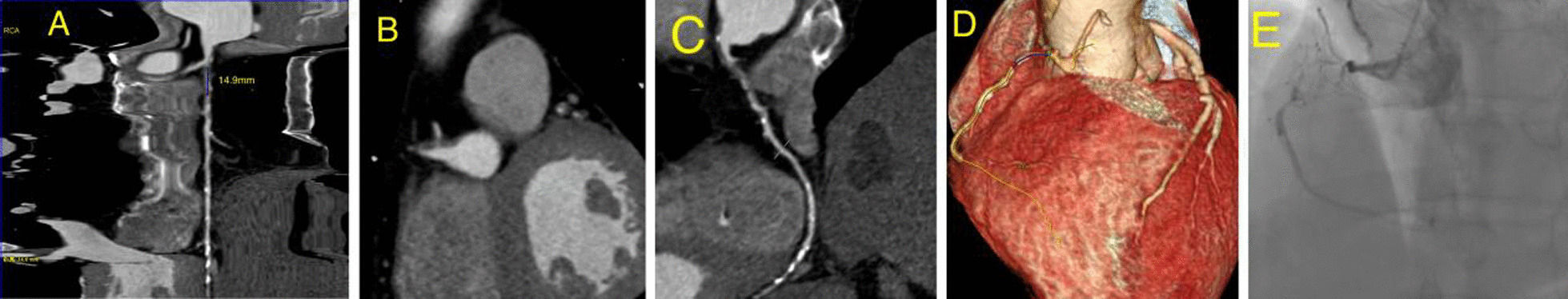
A CTO lesion in the proximal segment of RCA: without CABG history, age 57, Length ≤ 20 mm, tapered stump, non-severe calcification, non-severely tortuosity. **A**–**D** The CASTLE_CTA_ score was 0. **E** CASTLE_CAG_ score was 1

**Fig. 2:**
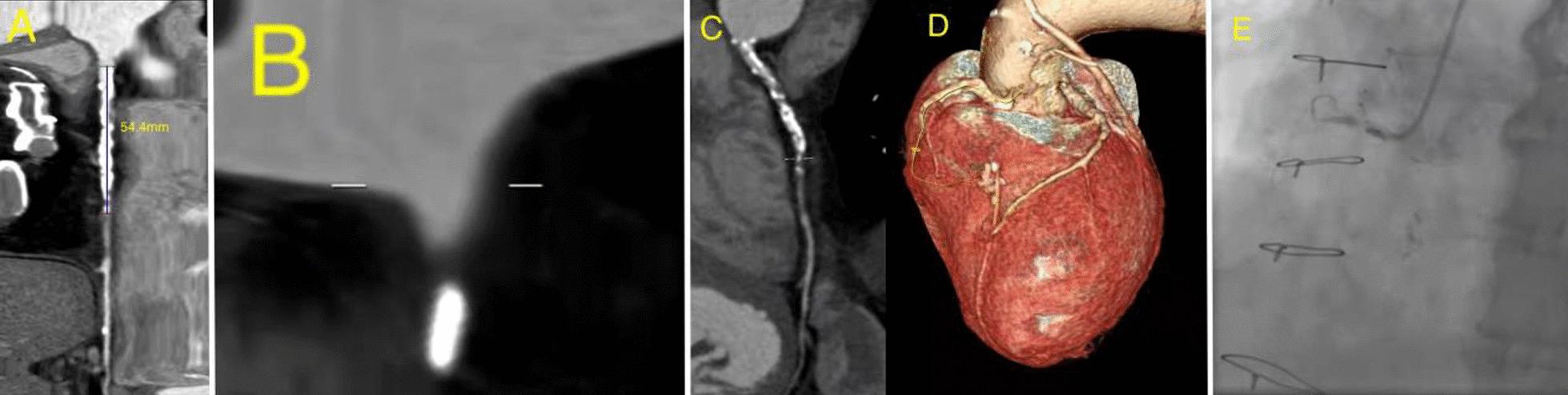
A CTO lesion in the proximal segment of RCA: with CABG history, age 77, Length > 20 mm, blunt stump, severe calcification, non-severely tortuosity. **A**–**D** The CASTLE_CTA_ score was 5. **E** CASTLE_CAG_ score was 4

The median CASTLE_CAG_ scores (4 [IQR, 2.5–5.5] vs 3 [IQR, 2–3]; p = 0.013) and the median CASTLE_CTA_ scores (4 [IQR, 3–5.5] vs 3 [IQR, 2–4]; p = 0.035) were significantly higher in patients in the procedural failure group than in the procedural success group. There was no significant difference in the median CASTLE_CTA_ and CASTLE_CAG_ scores in the procedural success group (3 [IQR, 2–4] vs 3 [IQR, 2–3]; p = 0.126). However, the median CASTLE_CTA_ score was significantly higher than the median CASTLE_CAG_ score among patients in the procedural failure group (4 [IQR, 3–5.5] vs 4 [IQR, 2.5–5.5]; p = 0.021). The median CASTLE_CAG_ scores (3 [IQR, 2–4] vs 2 [IQR, 2–3]; p < 0.001) and the median CASTLE_CTA_ scores (3 [IQR, 3–4] vs 3 [IQR, 2–3]; p < 0.001) were significantly higher in patients in whom wire crossing within 30 min failed than in those in whom wire crossing within 30 min was successful. There were no significant differences in the CASTLE_CTA_ and CASTLE_CAG_ scores among patients in whom wire crossing within 30 min failed (3 [IQR, 3–4] vs 3 [IQR, 2–4]; p = 0.254). However, the median CASTLE_CTA_ score was significantly higher than the median CASTLE_CAG_ score in the successful 30-min wire crossing group (3 [IQR, 2–3] vs 2 [IQR, 2–3]; p < 0.001). The median CASTLE_CAG_ score (3 [IQR, 3–4] vs 2 [IQR, 1–2]; p < 0.001) and the median CASTLE_CTA_ score (3.5 [IQR, 3–4] vs 1 [IQR, 0–2]; p < 0.001) were significantly higher in patients in the 30-min wire crossing failure group with antegrade wire access than in those in the 30-min wire crossing success group. There was no significant difference in the median CASTLE_CTA_ and CASTLE_CAG_ score among patients in the 30-min wire crossing failure group with antegrade wire access (3.5 [IQR, 3–4] vs 3 [IQR, 3–4]; p = 0.166). However, the median CASTLE_CTA_ score of patients in the 30-min wire crossing group with antegrade wire access was significantly lower than the median CASTLE_CAG_ score (1 [IQR, 0–2] vs 2 [IQR, 1–2]; p < 0.001). The median CASTLE_CAG_ score (3 [IQR, 3–4] vs 2 [IQR, 1.75–2.25]; p < 0.001) and the median CASTLE_CTA_ score (4 [IQR, 3–4] vs 2 [IQR, 1–2.5]; p < 0.001) were significantly higher in patients in whom wire crossing within 30 min failed with hybrid than in those in whom 30-min wire crossing was successful. There was no significant difference in the median CASTLE_CTA_ score and the median CASTLE_CAG_ score in the 30-min wire crossing group with hybrid (2 [IQR, 1–2.5] vs 2 [IQR, 1.75–2.25]; p = 0.914]. However, the median CASTLE_CTA_ score was significantly higher than the median CASTLE_CAG_ score in the 30-min guidewire crossing failure group with hybrid (4 [IQR, 3–4] vs 3 [IQR, 3–4]; p < 0.001) (Table 3, Figs. 1, 2).

**Table 3:** Effect of CASTLE scores derived on CAG versus CCTA on procedural results

	CAG-Derived	CCTA-Derived	***P***
Procedural			
Success	3(2–3)	3 (2–4)	0.126
Failure	4 (2.5–5.5)	4 (3–5.5)	P = 0.021
*P*	0.013	0.035	
30-min crossing			
Success	2 (2–3)	3(2–3)	**p < 0.001**
Failure	3 (2–4)	3(3–4)	0.254
*P*	p < 0.001	p < 0.001	
Antegrade			
< 30-min crossing	2 (1–2)	1 (0–2)	**p < 0.001**
> 30-minutecrossing)	3 (3–4)	3.5(3–4)	**0.166**
*P*	p < 0.001	p < 0.001	
Hybird			
< 30-min crossing	2 (1.75–2.25)	2 (1–2.5)	**0.914**
> 30-minutecrossing)	3 (3–4)	4 (3–4)	**0.001**
*P*	p < 0.001	p < 0.001	

CAG, coronary angiography; CCTA, coronary computed tomography angiography

### Comparison of CASTLE scores derived from CCTA and CAG for CTO lesions

The median CASTLE_CTA_ score was significantly higher than the median CASTLE_CAG_ score (3 [IQR, 2–4] vs 3 [IQR, 2–3]; p = 0.004). The CASTLE_CTA_ score described higher calcification levels than the CASTLE_CAG_ score (48.1% vs 33.8%; p = 0.015). However, there was no significant difference between the two groups of scores in stump anatomy, tortuosity degree, or occlusion length (≥ 20 mm) (all p > 0.05) (Table 4).

**Table 4:** CASTLE scores derived on CAG versus CCTA in CTO lesions

	CAG-Derived	CTA-Derived	*P*
CASTLE score	3 (2–3)	3 (2–4)	0.004
Stump anatomy [blunt or invisible]	116 (75.3)	108 (70.1)	0.371
Tortuosity degree [severe or unseen]	41 (26.6)	46 (29.9)	0.613
Length of occlusion [≥ 20 mm]	88 (57.1)	96 (62.3)	0.416
Extent of calcification [severe]	52 (33.8)	74 (48.1)	0.015

Data are presented as median (interquartile range) or number (%)

CAG, coronary angiography; CCTA, coronary computed tomography angiography; CTO, chronic total occlusion

### Prediction accuracy of CASTLE scores derived on CCTA and CAG

**Fig. 3:**
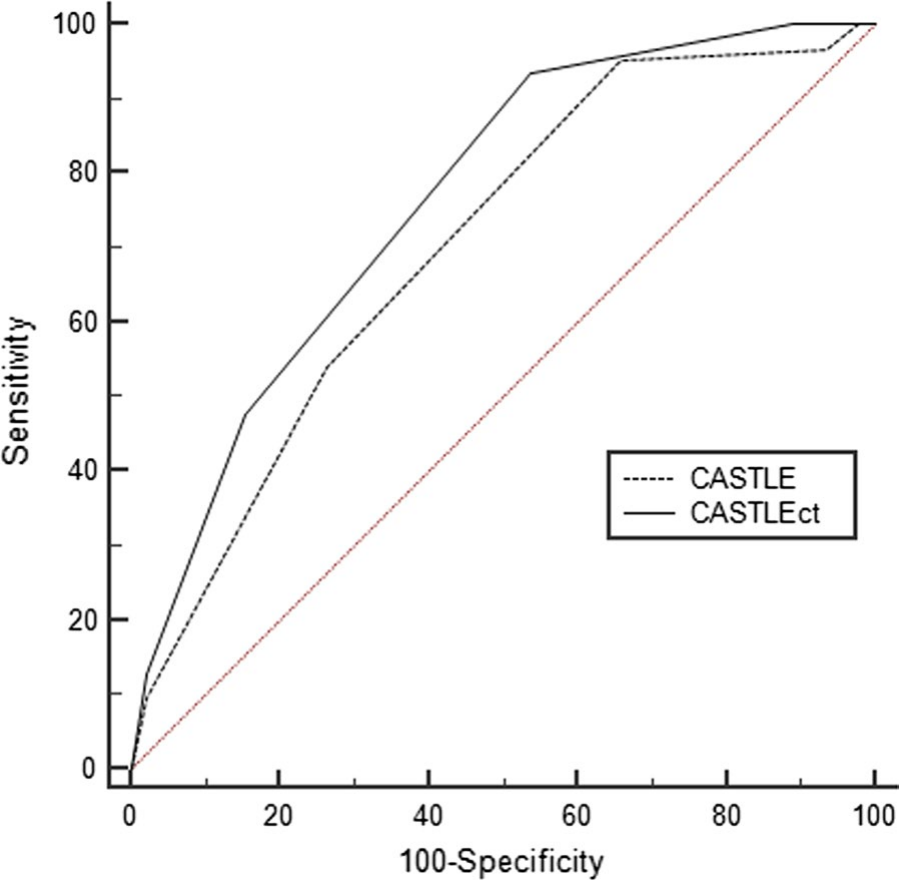
Receiver operating characteristic curves of coronary computed tomography angiography–derived CASTLE score for predicting < 30-min guidewire crossing success

**Fig. 4:**
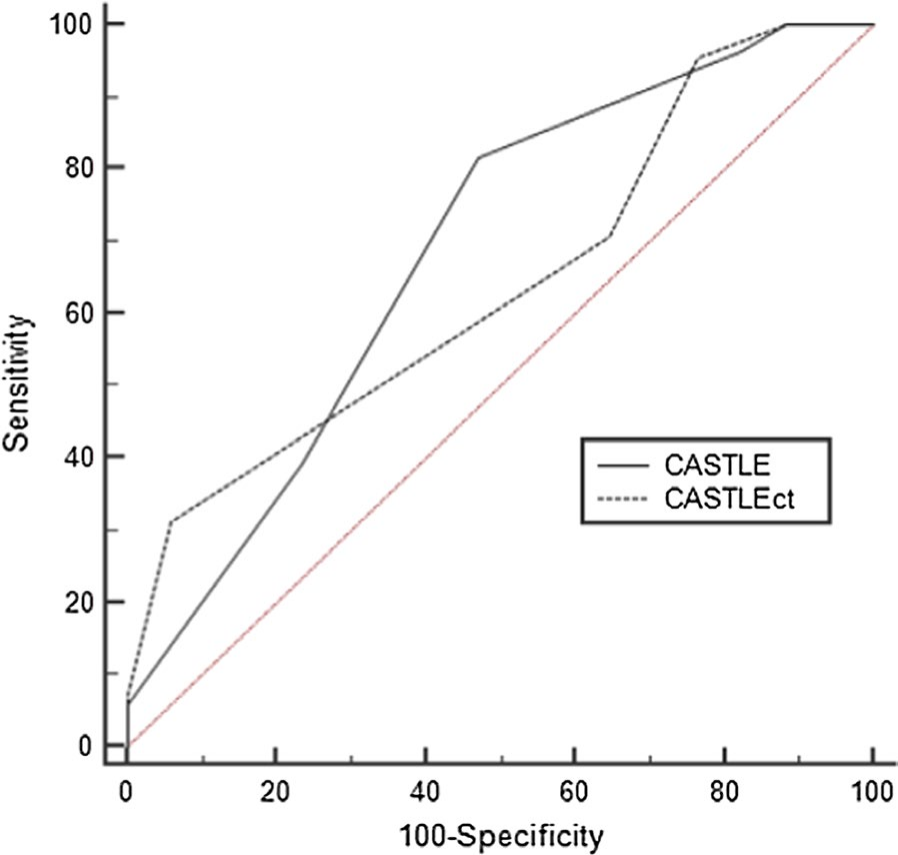
Receiver operating characteristic curves of coronary computed tomography angiography–derived CASTLE scores for predicting procedural success

In terms of predicting procedural success, there was no significant difference between the CASTLE_CTA_ score (AUC, 0.643; 95% CI, 0.561–0.718) and the CASTLE_CAG_ score (AUC, 0.685; 95% CI, 0.606–0.758; p = 0.488). However, the CASTLE_CTA_ score (AUC, 0.744; 95% CI, 0.667–0.811) was significantly better than the CASTLE_CAG_ score (AUC, 0.681; 95% CI, 0.601–0.754) for predicting 30-min wire crossing of CTO-PCI (p = 0.046). In terms of predicting 30-min wire crossing success, the best cut-off value for CASTLE_CTA_ was ≤ 3. The sensitivity, specificity, positive predictive value, and negative predictive value of a CASTLE score ≤ 3 were 90.8%, 55.2%, 54.6%, and 87.0%, respectively (Figs. 3 and 4).

## Discussion

This study showed that the CASTLE_CTA_ score obtained on noninvasive CCTA had better predictive capacity than the CASTLE_CAG_ score obtained on coronary angiography for 30-min wire crossing but had similar predictive ability for CTO-PCI procedural success.

An increasing number of scholars have designed and developed multiple scoring systems to predict CTO-PCI difficulty based on CAG. However, some scoring systems were derived from a single institution, some were based on antegrade wire access only, and others had relatively small sample sizes. The EuroCTO (CASTLE) score [11], with the largest sample size available to date (20,627 patients from 55 European centers and each procedure performed by an operator with extensive experience), resulted in a score related to hybrid strategies that had good predictive capacity for the difficulty of procedures. Compared with the J-CTO score, the CASTLE score includes four imaging features, namely stump anatomy, tortuosity, occlusion length, and calcification extent, similar to the J-CTO score. Moreover, the CASTLE score included two additional objective variables: age and CABG history. In the CASTLE scoring study, the incidence of CABG was 13%, which helped explain why it was necessary to consider CABG as an important variable. In the previous major scoring systems, the incidence of CABG was 6–9%, while that in our study was 12.7%, which was one reason why we chose the CASTLE score for prediction. In contrast to the J-CTO score, the CASTLE score did not include previously failed attempts in the final prediction model. This parameter depended on operator expertise. Less experienced operators were more likely to fail simply because they have not mastered all CTO-PCI revascularization techniques and therefore had limited options when managing difficult CTO cases, whereas experienced operators may be successful on the first attempt. Thus, for operators experienced in CTO-PCI, a previously failed attempt did not necessarily mean a more complex procedure or a greater likelihood of procedural failure. Notably, reverse controlled antegrade and retrograde tracking (reverse-CART) has been the most commonly used retrograde crossing technique, Actually we aimed to study 30-min antegrade wire crossing between CAG-score and CTA-score, 30-min wire crossing was set as an endpoint because it has been a more historically objective index of procedural difficulty [8, 12–15]. In fact in our center 30 min of crossing time is not an easy work but it could be completed in some specific patients especially in patients with good collateral circulation. Significantly higher CASTLE scores (both CTA- and CAG-derived) for > 30-min guide wire crossing and final antegrade- and hybrid crossing were observed in the procedural failure group than in the success group, indicating the predictive capacity of the CASTLE score (both CTA- and CAG-derived) for procedural success and guidewire crossing within 30 min.

Fujino [13] et al. demonstrated the importance of CCTA, as a noninvasive adjunctive test, in assessing the preoperative features of CTO lesions and as a valid alternative to invasive angiography in certain indications. Fujino et al. also concluded that the J-CTO score derived from CCTA had better ability to predict procedural and 30-min wire crossing success, especially for complex lesions. Determining the location and morphology of the stump is key to selecting the best CTO-PCI strategy. Attempts to cross an ill-defined stump may lead to perforation, and retrograde approach is usually recommended as the primary strategy if the stump’s anatomical ambiguity cannot be resolved. Therefore, accurate stump identification is extremely important to the choice of procedural strategy. CCTA distinguishes between obtuse and tapered stumps and can be used to determine the exact origin and direction of the side branch. In addition to assessing CTO entrance shape, CCTA more clearly shows the features related to the proximal cap, which are not readily available via any other imaging assessment tools. Rolf et al. [16] reported that CTA had superior ability to detect a blunt stump than conventional angiography because the time needed for CCTA may result in the retention of contrast medium in the microchannel within the CTO segment. However, we did not find that CASTLE_CCTA_ was superior to CASTLE_CAG_ for identifying blunt stumps.

Calcification length and severity were proportional to coronary occlusion duration, which increased CTO-PCI difficulty [17]. Calcification at the proximal (but not distal) fibrous cap was a major obstacle to successful guidewire crossing using an antegrade entry procedural strategy. Therefore, the identification of calcification length and severity was critical for guidewire and microcatheter selection, planning of guidewire upgrading and downgrading strategies, and knuckle technique guidance. CCTA was more sensitive for identifying, locating, and quantifying calcification than CAG [18, 19]. Unlike the J-CTO score, the CASTLE score assigned a score of 1 to severe calcification (extension of calcification to > 50% of the segment), which may avoid over- or underestimating the impact of calcification. In this study, the CASTLE_CTA_ score was better than the CASTLE_CAG_ score for assessing calcification.

CTO tortuosity had an important impact on guidewire selection. For significantly tortuous CTO lesions (especially long lesions), polymer-jacketed guidewires are preferred, while rigid guidewires may be used for straight and short CTO segments [20], which may be an area worth investigating in the future. In addition, CTA clearly identified and accurately quantified coronary tortuosity, which was often underestimated on CAG [8, 9]. The J-CTO score assigned a score of 1 to bending > 45°, while the CASTLE score graded tortuosity: straight (the pre-occlusive segment contained a bend of < 70°); moderate (a segment containing 2 bends > 70° or 1 bend > 90°); severe (CTO vessel contained 2 or more pre-occlusive bends > 90° or at least 1 bend > 120°). Tortuosity degree (severe or unseen) was assigned a score of 1, which could also avoid over- or underestimating the effect of tortuosity. CCTA are prone to detect angiographically invisible segments, nevertheless, there were no differences in terms of tortuosity in between the CCTA and CAG groups in our study, and it was presumed that a significant proportion of patients had ipsilateral circulation or required contralateral angiography guidance.

It is worth noting that preoperative CCTA has many disadvantages, such as higher cost, more radiation, and more contrast, etc. However, the flaws do not hide the beauty, in terms of potential advantages, noninvasive CCTA can not only detect CTO but also identify coronary lesions that may progress CTO. Specifically, a minimum lumen diameter < 2.0 mm, a reference segment diameter < 3.2 mm, and a mean plaque attenuation < 50 Hounsfield units on CTA were reported as independent predictors of future CTO lesions [21, 22], furthermore, a randomized trial [23] showed that the success rate of CTO-PCI guided by preoperative coronary CTA was higher, the operation or fluoroscopy time was not significantly prolonged, the dosage of contrast medium was not significantly increased, and the perioperative complications (such as coronary artery perforation or perioperative myocardial infarction) were less, which mean that CCTA may help to prevent serious complications during CTO-PCI. Therefore, coronary CTA may be an indispensable part of safe and effective CTO-PCI, although the low incidence of complications in that study needs more randomized studies to prove.

### Limitations

This study has several limitations. First, it was a single-center retrospective observational study with a relatively small sample size, and its results require further confirmation by a prospective multicenter study. Second, the lesion information provided by preoperative CTA may contribute to procedural success and guidewire crossing within 30 min. Third, only CTO-PCI patients who underwent preoperative contrast-enhanced CTA were selectively included. Data on excluded patients were not available because our study protocol was only applicable to the final study population.

## Conclusion

The CASTLE_CTA_ score obtained on noninvasive CCTA was better at predicting 30-min guidewire crossing success than the CASTLE_CAG_ score obtained on coronary angiography.
